# Supplementary material for: MetaRibo-Seq measures translation in microbiomes
Source: Nat Commun. 2020 Jun 29;11:3268. doi: 10.1038/s41467-020-17081-z (PMC7324362; doi:10.1038/s41467-020-17081-z)
Supplement: Supplementary file 10 — Supplementary Data 7 [file 41467_2020_17081_MOESM10_ESM.zip › File2/Confidence_High_Taxonomy/357215_out.krona.html]

Javascript must be enabled to view this page.

members
magnitude
magnitudeUnassigned
count
unassigned
taxon
rank

357215\_out

233

233
superkingdom
2

976
phylum
232

200643
class
232

order
232
171549

family
1
171550

1
genus
239759

species
1

SRS097920\_contig\_number\_8682
214856

815
family
38

38
genus
816

47678

SRS020233\_contig\_number\_37530SRS022137\_contig\_number\_17718SRS055982\_contig\_number\_16455SRS147346\_contig\_number\_8760
4
species


SRS018836\_contig\_number\_14861SRS064557\_contig\_number\_3212
2292944
species
2


SRS145308\_contig\_number\_4178
820
species
1

1638780

SRS014923\_contig\_number\_28356SRS016840\_contig\_number\_1825SRS018049\_contig\_number\_1722SRS023526\_contig\_number\_16492SRS023583\_contig\_number\_10002SRS052078\_contig\_number\_4400SRS054352\_contig\_number\_contig-100\_1447.136073SRS063985\_contig\_number\_31288SRS076876\_contig\_number\_661SRS101268\_contig\_number\_contig-100\_178.179SRS144135\_contig\_number\_9744SRS146812\_contig\_number\_61789SRS148159\_contig\_number\_7619SRS893252\_contig\_number\_4987
14
species

821

SRS142923\_contig\_number\_21915
species
1


SRS1055069\_contig\_number\_10405
371601
species
1

329854

SRS1041136\_contig\_number\_20707
species
1


SRS045713\_contig\_number\_10131SRS048981\_contig\_number\_3208SRS051882\_contig\_number\_10501SRS097889\_contig\_number\_16617
818
species
4

2
species

SRS063190\_contig\_number\_12772SRS104036\_contig\_number\_7444
817

338188

SRS063040\_contig\_number\_31321
species
1

species
1
2292938

SRS105153\_contig\_number\_25654


SRS019161\_contig\_number\_16641SRS075341\_contig\_number\_18987
246787
2
species

2
species
2292942

SRS045739\_contig\_number\_8251SRS074670\_contig\_number\_6502

1
species

SRS1054716\_contig\_number\_11043
46506


SRS143722\_contig\_number\_15220
1796613
1
species

1853231
2
family

2
283168
genus
1

SRS144714\_contig\_number\_27331

1
species
28118

SRS147139\_contig\_number\_25644

2005525
family
191

375288
191
genus

1
species

SRS014613\_contig\_number\_2943
563193

46503

SRS011302\_contig\_number\_22202SRS011405\_contig\_number\_11843SRS013098\_contig\_number\_34263SRS013521\_contig\_number\_12713SRS013800\_contig\_number\_3010SRS013940\_contig\_number\_21238SRS013951\_contig\_number\_contig-100\_247.208277SRS014235\_contig\_number\_12964SRS014287\_contig\_number\_18329SRS014412\_contig\_number\_16117SRS014415\_contig\_number\_contig-100\_176.177SRS014459\_contig\_number\_35320SRS014683\_contig\_number\_13968SRS014948\_contig\_number\_21006SRS015065\_contig\_number\_36384SRS015264\_contig\_number\_20177SRS015431\_contig\_number\_37426SRS015663\_contig\_number\_28326SRS015694\_contig\_number\_10628SRS015782\_contig\_number\_40739SRS016018\_contig\_number\_13640SRS016056\_contig\_number\_5407SRS016132\_contig\_number\_4355SRS016381\_contig\_number\_contig-100\_90.34928SRS016753\_contig\_number\_4934SRS017247\_contig\_number\_3330SRS017433\_contig\_number\_13252SRS017521\_contig\_number\_45821SRS017701\_contig\_number\_19406SRS018133\_contig\_number\_6505SRS018888\_contig\_number\_2246SRS018936\_contig\_number\_11188SRS019030\_contig\_number\_14222SRS019068\_contig\_number\_27611SRS019178\_contig\_number\_contig-100\_907.908SRS019286\_contig\_number\_5769SRS019381\_contig\_number\_15212SRS019397\_contig\_number\_9005SRS019445\_contig\_number\_29888SRS019685\_contig\_number\_39408SRS019808\_contig\_number\_18599SRS019910\_contig\_number\_23271SRS020271\_contig\_number\_378SRS020328\_contig\_number\_21686SRS022524\_contig\_number\_15450SRS023914\_contig\_number\_24330SRS024009\_contig\_number\_4686SRS024075\_contig\_number\_11137SRS024435\_contig\_number\_17733SRS024492\_contig\_number\_contig-100\_1176.105865SRS042628\_contig\_number\_28383SRS042966\_contig\_number\_19190SRS043667\_contig\_number\_contig-100\_651.25518SRS043841\_contig\_number\_10774SRS044535\_contig\_number\_15652SRS045004\_contig\_number\_24456SRS045244\_contig\_number\_3072SRS045645\_contig\_number\_16945SRS046068\_contig\_number\_1216SRS046369\_contig\_number\_12593SRS047044\_contig\_number\_7208SRS047433\_contig\_number\_18556SRS048164\_contig\_number\_24010SRS048870\_contig\_number\_contig-100\_734.132856SRS049402\_contig\_number\_5621SRS049712\_contig\_number\_22681SRS049773\_contig\_number\_26089SRS049896\_contig\_number\_21274SRS049959\_contig\_number\_42603SRS049995\_contig\_number\_24697SRS050026\_contig\_number\_9736SRS050422\_contig\_number\_34069SRS050520\_contig\_number\_649SRS050925\_contig\_number\_10224SRS050941\_contig\_number\_16309SRS051031\_contig\_number\_34726SRS051610\_contig\_number\_11719SRS052697\_contig\_number\_44659SRS053356\_contig\_number\_42497SRS053398\_contig\_number\_13972SRS053649\_contig\_number\_21658SRS054905\_contig\_number\_23672SRS055966\_contig\_number\_109SRS058070\_contig\_number\_22456SRS058770\_contig\_number\_25843SRS062427\_contig\_number\_3874SRS062654\_contig\_number\_25489SRS063127\_contig\_number\_2734SRS063370\_contig\_number\_4119SRS063489\_contig\_number\_5940SRS064232\_contig\_number\_4011SRS064276\_contig\_number\_40616SRS064757\_contig\_number\_20468SRS065176\_contig\_number\_10986SRS065397\_contig\_number\_9898SRS065504\_contig\_number\_contig-100\_692.211167SRS075021\_contig\_number\_6330SRS075821\_contig\_number\_20431SRS075984\_contig\_number\_6893SRS076804\_contig\_number\_17593SRS076976\_contig\_number\_17843SRS077024\_contig\_number\_11601SRS077086\_contig\_number\_11376SRS077231\_contig\_number\_15682SRS077294\_contig\_number\_11738SRS077552\_contig\_number\_7026SRS077589\_contig\_number\_3709SRS077641\_contig\_number\_19814SRS077849\_contig\_number\_22661SRS078176\_contig\_number\_18863SRS078242\_contig\_number\_21412SRS078419\_contig\_number\_33189SRS078665\_contig\_number\_7388SRS097958\_contig\_number\_21670SRS098061\_contig\_number\_9567SRS098571\_contig\_number\_59336SRS098644\_contig\_number\_8817SRS098655\_contig\_number\_contig-100\_468.94571SRS101376\_contig\_number\_11743SRS103987\_contig\_number\_3912SRS1041039\_contig\_number\_28147SRS1041091\_contig\_number\_3018SRS1041092\_contig\_number\_15933SRS1041112\_contig\_number\_5199SRS1041116\_contig\_number\_6149SRS1041118\_contig\_number\_15956SRS1041130\_contig\_number\_4780SRS1041134\_contig\_number\_13623SRS1041137\_contig\_number\_21292SRS1041138\_contig\_number\_2734SRS1041139\_contig\_number\_2291SRS1041140\_contig\_number\_21160SRS1041141\_contig\_number\_13356SRS104311\_contig\_number\_22062SRS104327\_contig\_number\_contig-100\_43.25901SRS104636\_contig\_number\_27008SRS104912\_contig\_number\_contig-100\_77.120000SRS104975\_contig\_number\_21037SRS105082\_contig\_number\_17906SRS1055022\_contig\_number\_18630SRS140492\_contig\_number\_28481SRS140513\_contig\_number\_13287SRS140513\_contig\_number\_13722SRS142503\_contig\_number\_6442SRS142542\_contig\_number\_contig-100\_1491.149214SRS143070\_contig\_number\_10351SRS143148\_contig\_number\_24452SRS143181\_contig\_number\_13660SRS143372\_contig\_number\_9019SRS143466\_contig\_number\_3295SRS143780\_contig\_number\_23965SRS143876\_contig\_number\_5796SRS143991\_contig\_number\_18958SRS144297\_contig\_number\_11912SRS144506\_contig\_number\_44951SRS144537\_contig\_number\_42981SRS144603\_contig\_number\_16954SRS145497\_contig\_number\_19685SRS146764\_contig\_number\_15873SRS146813\_contig\_number\_17781SRS147271\_contig\_number\_15707SRS147425\_contig\_number\_2874SRS147557\_contig\_number\_24869SRS148091\_contig\_number\_14214SRS148253\_contig\_number\_12230SRS148424\_contig\_number\_27926SRS148721\_contig\_number\_51277SRS148784\_contig\_number\_25870SRS148817\_contig\_number\_13259SRS149075\_contig\_number\_14984SRS149181\_contig\_number\_26198SRS893170\_contig\_number\_8122SRS893230\_contig\_number\_904SRS893292\_contig\_number\_6803SRS893295\_contig\_number\_4173SRS893300\_contig\_number\_9143SRS893327\_contig\_number\_2501SRS893358\_contig\_number\_10369SRS893378\_contig\_number\_21084SRS893382\_contig\_number\_3396
180
species

10
species
1263094

SRS013476\_contig\_number\_41179SRS017821\_contig\_number\_5818SRS017859\_contig\_number\_2211SRS019968\_contig\_number\_14669SRS024132\_contig\_number\_49797SRS046717\_contig\_number\_9840SRS054059\_contig\_number\_4435SRS101433\_contig\_number\_12503SRS148196\_contig\_number\_37997SRS148319\_contig\_number\_13355

phylum
1
1239

186801
1
class

186802
1
order

186803
1
family

841
1
genus

species
1
2049040

SRS012969\_contig\_number\_34740
